# Early scar-directed interventions for thyroidectomy scars: a systematic review and network meta-analysis

**DOI:** 10.3389/fsurg.2026.1914326

**Published:** 2026-09-08

**Authors:** Kaiyi Yu, Zhuolun Hao, Jiaming Sun

**Affiliations:** 1 Department of Plastic Surgery, Union Hospital, Tongji Medical College, Huazhong University of Science and Technology, Wuhan, China; 2 Department of Plastic Surgery, Chongqing Hospital, Union Hospital, Tongji Medical College, Huazhong University of Science and Technology, Chongqing, China

**Keywords:** anterior neck scar, network meta-analysis, scar-directed intervention, thyroidectomy scar, wound healing

## Abstract

Visible anterior neck scars after thyroidectomy and related cervicotomy procedures remain clinically important aesthetic and psychosocial concerns. This systematic review and network meta-analysis compared randomized evidence for early scar-directed interventions, while separating surgical closure method trials from postoperative scar modulation strategies. PubMed/MEDLINE, Embase, and Cochrane CENTRAL were searched independently from database inception to 17 June 2026. The primary outcome was scar quality at the longest available 3- to 12-month follow-up, assessed using POSAS, OSAS/PSAS, VSS/mVSS, SBSES, and MSS in a prespecified hierarchy. The primary connected network included six randomized trials comprising 408 unique participants and 469 analyzed scar/incision observations, with 10 direct comparisons and eight scar-directed treatment nodes. Laser plus steroid demonstrated a favorable low-certainty network estimate (standardized mean difference (SMD) −1.35, 95% CI −1.87 to −0.83), as did laser/light-based protocols (SMD −0.95, 95% CI −1.40 to −0.50). Platelet-rich plasma also showed a favorable estimate (SMD −0.95, 95% CI −1.73 to −0.16), but this evidence was very uncertain because the node was supported by a single small study with methodological concerns. CINeMA certainty was low or very low across comparisons. Closure method trials were retained as a descriptive evidence map because their mechanisms, timing, and clinical decision context differed from scar-directed therapy. These findings should be interpreted as hypothesis-generating and do not support definitive comparative superiority claims.

**Systematic Review Registration:**

https://www.crd.york.ac.uk/prospero/display_record.php?ID=CRD420261427171, PROSPERO CRD420261427171.

## 1 Introduction

Visible anterior neck scars after thyroidectomy, parathyroidectomy, and related cervicotomy procedures can remain a source of dissatisfaction despite successful disease treatment (1). Scar erythema, hypertrophy, pigmentation, widening, contour irregularity, pruritus, and discomfort are particularly relevant in the anterior neck because the incision is exposed, mobile, and subject to transverse tension. Contemporary scar management recommendations emphasize prevention, tension control, inflammation modulation, and phenotype-specific treatment rather than delayed revision alone (2–4).

Early scar prevention is central to plastic, reconstructive, and aesthetic surgery because it targets wound biology before a mature scar deformity develops. Scar-directed interventions include neuromodulator injection, regenerative injection, silicone or tension modulation, and energy-based remodeling strategies. Evidence from broader scar literature supports biologically distinct intervention families, including silicone-based support, botulinum toxin type A, and laser-based remodeling, but these data are dispersed across scar types and anatomical sites (5–8).

Most trials compare only one or two strategies, while clinical decisions require comparison across multiple intervention families. A frequent source of confusion is that intraoperative closure techniques and postoperative scar-directed therapies both influence scar appearance but answer different clinical questions. Closure methods address how the incision is approximated during the operation; scar-directed therapies address how early postoperative inflammation, collagen remodeling, pigmentation, vascularity, or tension are modulated during scar maturation. Network meta-analysis can synthesize direct and indirect evidence when populations, timing, comparators, and outcome constructs are sufficiently comparable. However, sparse networks should not be interpreted as treatment hierarchies. The aim of this review was therefore to estimate comparative effects with certainty ratings and clarify which evidence belongs in the primary scar-directed network, rather than naming a definitive best intervention.

## 2 Materials and methods

### 2.1 Review design and reporting framework

This article was structured as a systematic review and network meta-analysis of randomized evidence, following PRISMA-NMA and PRISMA 2020 principles (9, 10). Before quantitative synthesis, studies were assigned to mechanistic analytical layers. Closure method trials were defined as interventions primarily determining wound-edge approximation during surgery, including closure materials, suture or staple technique, tissue adhesive, and related operative decisions. Early scar-directed therapies were defined as interventions intended to modulate postoperative scar biology, including inflammation, collagen remodeling, vascularity, pigmentation, pliability, thickness, or tension. This separation was used to preserve clinical transitivity and avoid mixing operative closure decisions with postoperative scar modulation decisions. The review was retrospectively registered in PROSPERO (CRD420261427171), with registration completed after the initial search and before final manuscript revision.

### 2.2 Information sources and search strategy

PubMed/MEDLINE, Embase, and the Cochrane Central Register of Controlled Trials (CENTRAL) were searched independently from database inception to 17 June 2026. Additional sources included PMC/open full-text retrieval, DOI/Crossref landing-page verification, ClinicalTrials.gov, WHO ICTRP, citation tracking from relevant systematic reviews, and manual checking of reference lists from eligible trials. Complete database-specific search strings—including PubMed syntax, Embase field tags/Emtree trial filter terms, and CENTRAL keyword syntax—are provided in Supplementary Table S1.

### 2.3 Eligibility criteria and study selection

Eligible studies included randomized controlled trials, randomized split-scar or split-incision studies, and randomized multiarm trials enrolling patients undergoing thyroidectomy, parathyroidectomy, cervicotomy, or clinically comparable anterior neck surgical incisions. The primary network was restricted to early scar-directed interventions intended to modulate inflammation, collagen remodeling, scar thickness, vascularity, pigmentation, tension, or patient/observer scar appearance. Surgical closure method trials were not combined with postoperative scar-directed interventions because closure methods address wound-edge approximation during operation rather than postoperative scar modulation. The mechanistic separation of these layers is illustrated in Supplementary Figure S9.

### 2.4 Outcome hierarchy and intervention nodes

The primary endpoint was scar quality at the longest available 3- to 12-month follow-up. This window was chosen because it captures early-to-intermediate scar maturation, when erythema, thickness, pliability, pigmentation, and patient-perceived scar appearance are still modifiable but sufficiently developed for scar assessment. Outcome scales were prioritized as POSAS, OSAS/PSAS, VSS/mVSS, SBSES, and MSS. These scales are not identical: POSAS emphasizes patient and observer scar perception, VSS/mVSS emphasizes objective scar properties, and SBSES/MSS emphasize wound cosmesis (11, 12). Therefore, standardized mean differences were used, and outcome-scale differences were considered in the transitivity assessment and CINeMA indirectness judgments. Primary treatment nodes were grouped as botulinum toxin type A, laser/light-based protocol, laser plus intralesional steroid protocol, platelet-rich plasma, polydeoxyribonucleotide (PDRN)/polynucleotide (PN), silicone dressing/sheet, elastic or tension-modulating silicone, and standard care/placebo/sham. This node construction intentionally favored clinical interpretability over excessive intervention fragmentation.

### 2.5 Assessment of transitivity and effect modifiers

Transitivity was assessed before quantitative synthesis by comparing potential effect modifiers across treatment nodes. The assessment considered surgical site and incision type, scar phenotype, timing of intervention initiation, follow-up window, outcome scale, comparator context, trial design, and population characteristics. The primary network was considered clinically defensible because all included studies addressed early scar prevention or early scar modulation for linear anterior neck surgical scars. However, differences in scar scales (POSAS, PSAS/OSAS, VSS/mVSS, SBSES, and MSS), follow-up timing from 3 to 12 months, and small single-center trial designs were treated as sources of clinical heterogeneity and indirectness rather than ignored. A structured transitivity assessment is provided in Supplementary Table S2.

### 2.6 Data extraction and risk of bias

Titles and abstracts were screened independently by KY and ZH. Potentially eligible full texts were then assessed independently by the same two reviewers according to predefined eligibility criteria. Disagreements were resolved by discussion, and unresolved discrepancies were adjudicated by JS. KY extracted study-level and arm-level data using a prespecified form, including study identifier, PMID/DOI, population, design, intervention and comparator details, sample size, follow-up time, scar scale, mean, standard deviation, adverse events where available, and data source. ZH independently checked numerical outcome data, follow-up selection, intervention node assignment, and study design classification against the original reports. RoB 2 and CINeMA certainty judgments were performed independently by KY and ZH and finalized by consensus using established methodological frameworks (13, 14).

### 2.7 Statistical analysis

For each analysis layer (Table 1), one scar-quality outcome per study was selected using the outcome hierarchy and the longest available follow-up. For denominator reporting, unique participants were counted once per study, whereas randomized split-scar or split-incision designs were additionally summarized at the scar/incision observation level used for the network model. Standardized mean differences were analyzed using a frequentist random-effects network meta-analysis implemented in R with the netmeta package and the meta package (15). Negative SMDs favored the scar-directed intervention. Unless otherwise specified, reported SMDs and 95% confidence intervals are random-effects network estimates rather than direct pairwise estimates. Because a common minimal clinically important difference is not available across POSAS, VSS/mVSS, SBSES, PSAS/OSAS, and MSS, SMDs were anchored using conventional approximate magnitudes—0.2 SD as small, 0.5 SD as moderate, and 0.8 SD or greater as large—while emphasizing that clinical importance remains scale- and context-dependent. Additional clinical anchoring of standardized mean difference (SMD) magnitudes is provided in Supplementary Table S3. For split-scar or split-incision trials, paired contrasts were handled by accounting for within-participant correlation. When paired standard deviations were unavailable, the standard error of the standardized contrast was reconstructed using an assumed within-participant correlation; the primary analysis used *r* = 0.50, and sensitivity analyses used *r* = 0.25 and *r* = 0.75. The full correlation sensitivity analysis is reported in Supplementary Table S4. Because the network was sparse, treatment-ordering metrics were not used to support superiority claims. Node-splitting and design-by-treatment tests were interpreted as exploratory assessments rather than definitive tests of network consistency (16, 17). Publication bias and small-study effects were not formally assessed because the primary network contained only six RCTs.

**Table 1:** Analysis layers and interpretation.

Analytical layer	Clinical question	Main mechanism/timing	Evidence handling	Interpretation
Primary scar-directed NMA	How can early postoperative scar maturation be modulated after thyroidectomy/anterior neck incision?	Postoperative or perioperative modulation of inflammation, collagen remodeling, vascularity/pigmentation, pliability, thickness, and tension	Six RCTs; 408 unique participants; 469 analyzed scar/incision observations; eight treatment nodes	Core analysis for scar-directed interventions; estimates interpreted with CINeMA certainty and without definitive treatment hierarchy
Disconnected eligible scar outcome trial	Does an eligible scar outcome comparison connect to the scar-directed evidence network?	Eligible scar outcome but closure comparison nodes were not connected to the scar-directed component	Retained descriptively; not forced into the primary connected model	Prevents artificial network connection and preserves interpretability
Closure method evidence map	How should the incision be approximated at the time of operation?	Intraoperative wound-edge apposition, closure material, track marks, early mechanical support, operative time, and surgeon preference	Moved to Supplementary Material as a descriptive evidence map	Not pooled with scar-directed therapies because timing, mechanism, and decision context differ
Sensitivity analyses	Are key assumptions robust?	Paired-data assumptions, PRP single-study node, source verification, and certainty considerations	Reported as supplementary sensitivity analyses where appropriate	Used to test robustness, not to create a comparative hierarchy

## 3 Results

### 3.1 Study selection and evidence base

**Figure 1:**
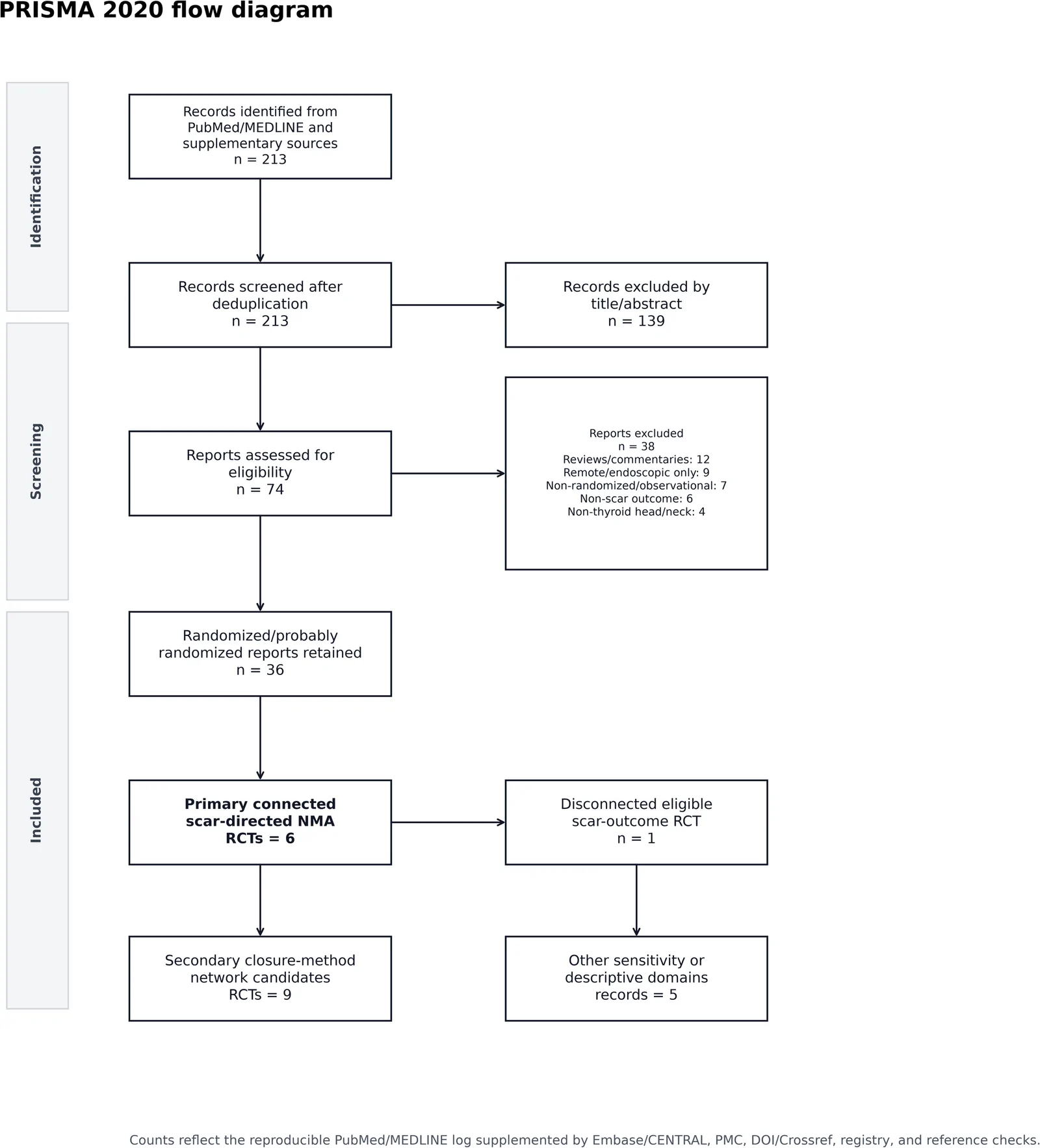
PRISMA flow diagram and analysis allocation.

The study-selection process and analysis allocation are shown in Figure 1. After screening, 36 randomized or potentially randomized records were retained for evidence mapping. Six RCTs formed the primary connected scar-directed network. The primary network included 408 unique participants and 469 analyzed scar/incision observations, with 10 direct comparisons and eight treatment nodes. One eligible RCT was retained descriptively because it was disconnected from the analyzable scar-directed network, and closure method studies were moved to the Supplementary Material as a separate evidence map.

### 3.2 Primary connected scar-directed network

The primary connected network was built from randomized trials evaluating botulinum toxin type A, laser/light-based protocol, laser plus steroid protocol, platelet-rich plasma (PRP), PDRN/PN injection, silicone dressing, tension-modulating silicone, and standard care/placebo/sham (18–23). A formal transitivity assessment was performed before pooling. The principal clinical commonality was that all primary-network trials addressed early scar prevention or early scar modulation for linear anterior neck surgical scars. The main threats to transitivity were heterogeneous scar scales, follow-up windows from 3 to 12 months, split-scar design features, and limited replication within nodes. These issues were incorporated into CINeMA indirectness/imprecision judgments and are summarized in Supplementary Table S2.

### 3.3 Risk of bias

**Figure 2:**
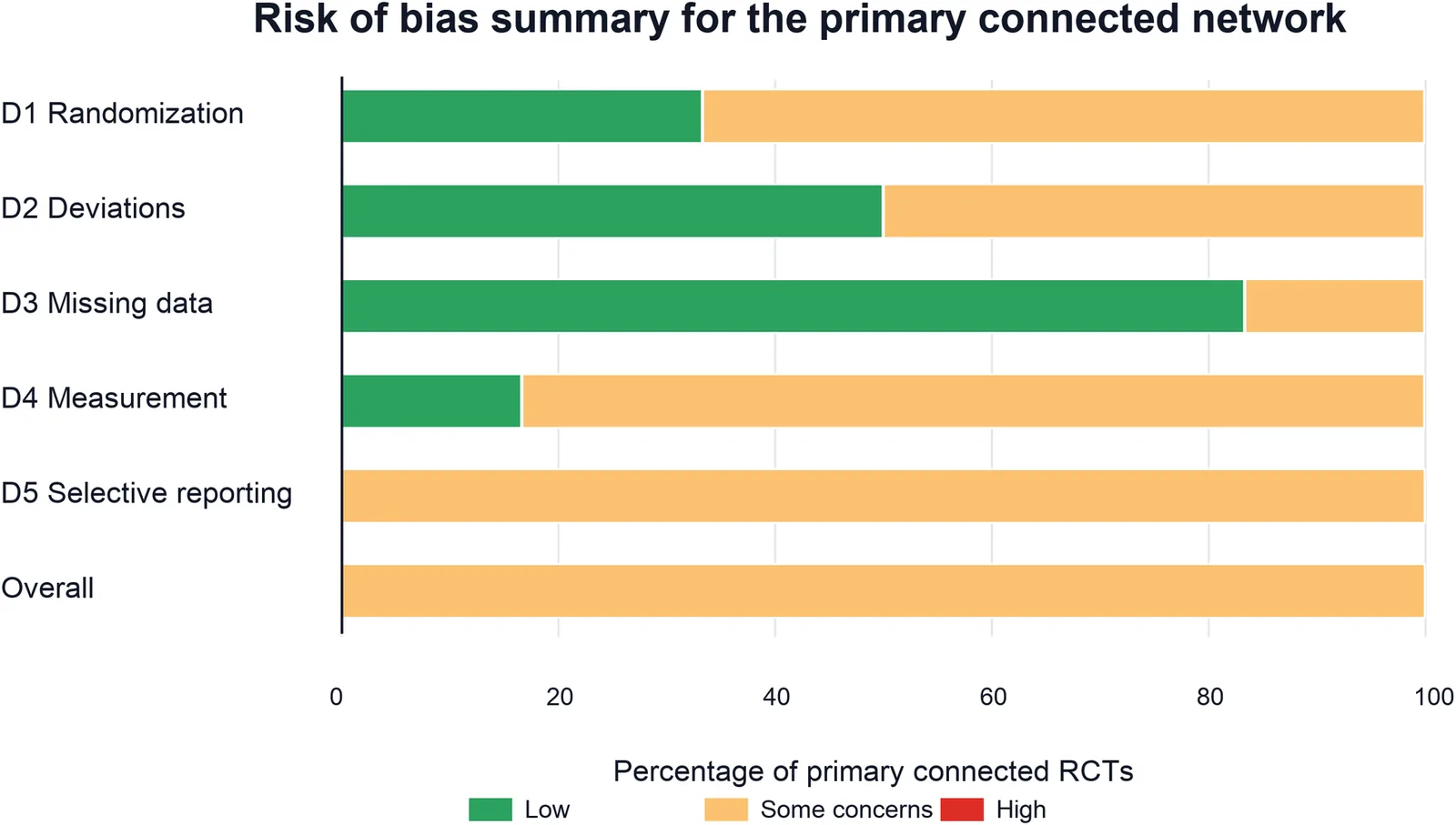
RoB 2 summary for the primary connected network.

Risk of Bias 2 judgments were conservative and based on the actual design, conduct, and reporting of each trial rather than database indexing labels. The most frequent overall judgment was some concerns, mainly because several trials had limited reporting of allocation concealment, protocol registration, assessor masking, outcome-reporting safeguards, or paired data handling for split-scar designs. The PRP study was revised to some concerns rather than high risk of bias: The full text described randomization, a concurrent control group, thyroidectomy scars, and blinded scar assessment, but concerns remained regarding incomplete reporting of participant/personnel blinding, prospective protocol availability, and prespecified outcome-reporting safeguards. The RoB 2 summary is shown in Figure 2.

Domain-level traffic-light judgments are provided in Supplementary Figure S1.

### 3.4 Network geometry

**Figure 3:**
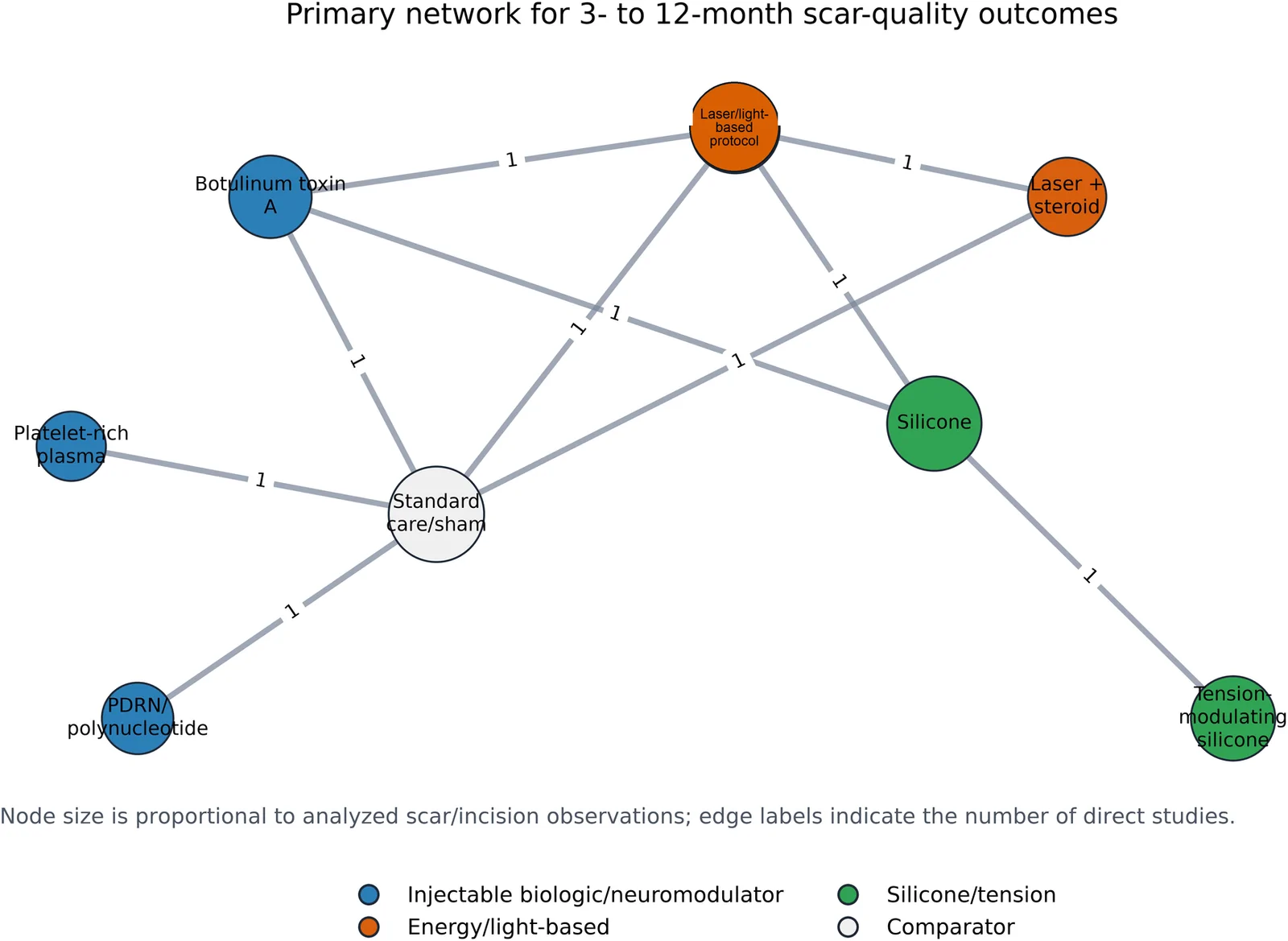
Primary connected scar-directed network geometry.

The primary 3- to 12-month connected scar-directed network included six randomized trials comprising 408 unique participants and 469 analyzed scar/incision observations, with 10 direct comparisons and eight treatment nodes. Characteristics of the primary connected studies are shown in Table 2. Node size in Figure 3 is proportional to analyzed scar/incision observations, and edge labels indicate the number of direct studies. The network was clinically focused but statistically sparse, with most nodes informed by a single study.

**Table 2:** Characteristics of studies included in the primary connected scar-directed network.

Study	PMID	Treatment nodes	Unique *N*; analyzed scar/incision obs.	Scale	Follow-up (months)
Bae et al. (18)	32245256	Botulinum toxin type A, standard care/placebo/sham	40; 40	SBSES	6
Chung et al. (19)	33567135	Laser/light-based protocol, laser plus intralesional steroid protocol, standard care/placebo/sham	126; 126	PSAS	6
Jiang et al. (20)	35852693	Silicone dressing/sheet, Elastic/tension-modulating silicone	61; 122	VSS	6
Kim et al. (21)	35713247	PDRN/polynucleotide injection, standard care/placebo/sham	44; 44	mVSS	3
Ren et al. (22)	41882220	Botulinum toxin type A, laser/light-based protocol, silicone dressing/sheet	105; 105	POSAS	12
Pk and Chaudhary (23)	40621269	Platelet-rich plasma injection, standard care/placebo/sham	32; 32	POSAS patient total	4

### 3.5 Primary network meta-analysis

**Figure 4:**
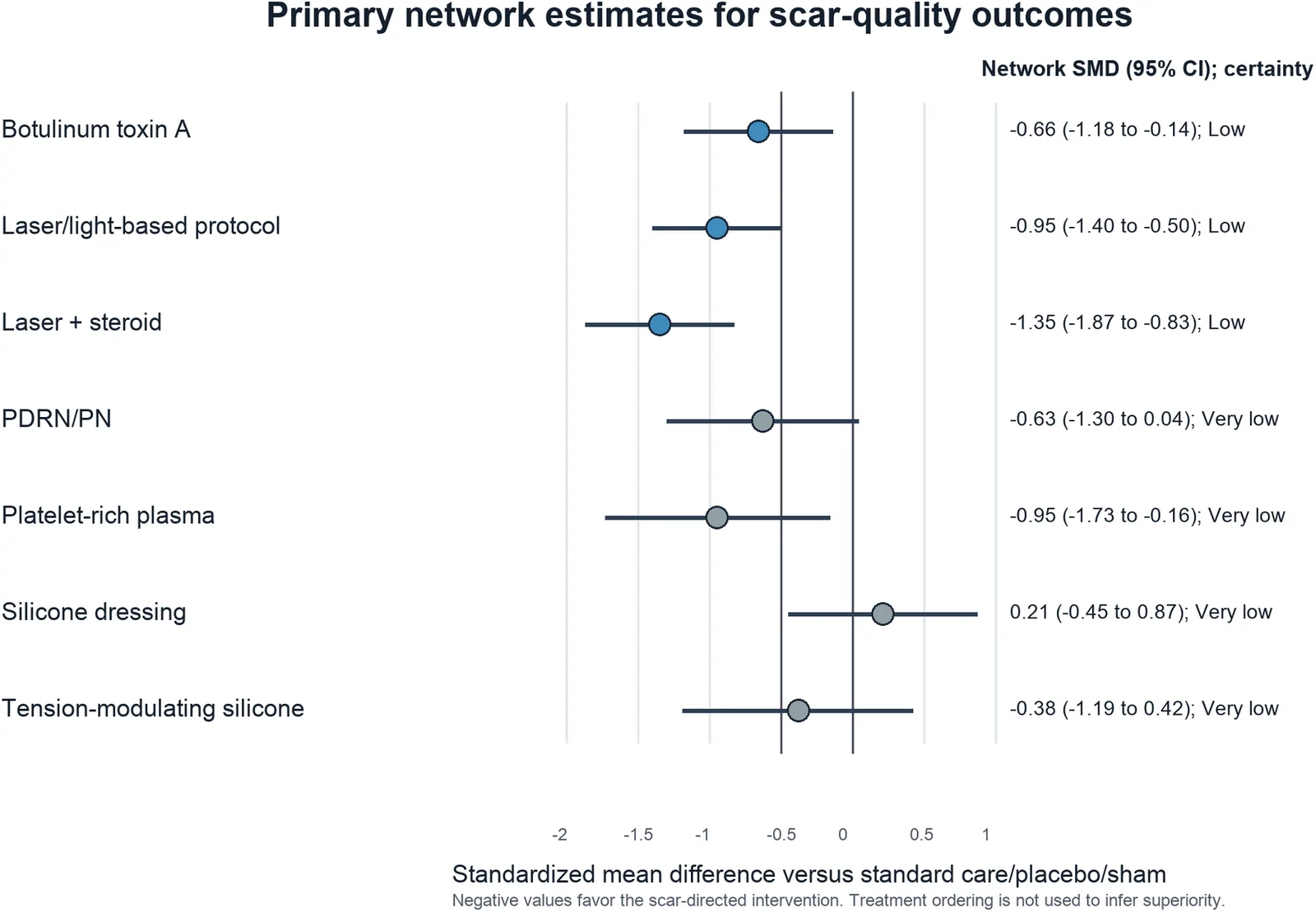
Forest plot of primary network estimates.

**Figure 5:**
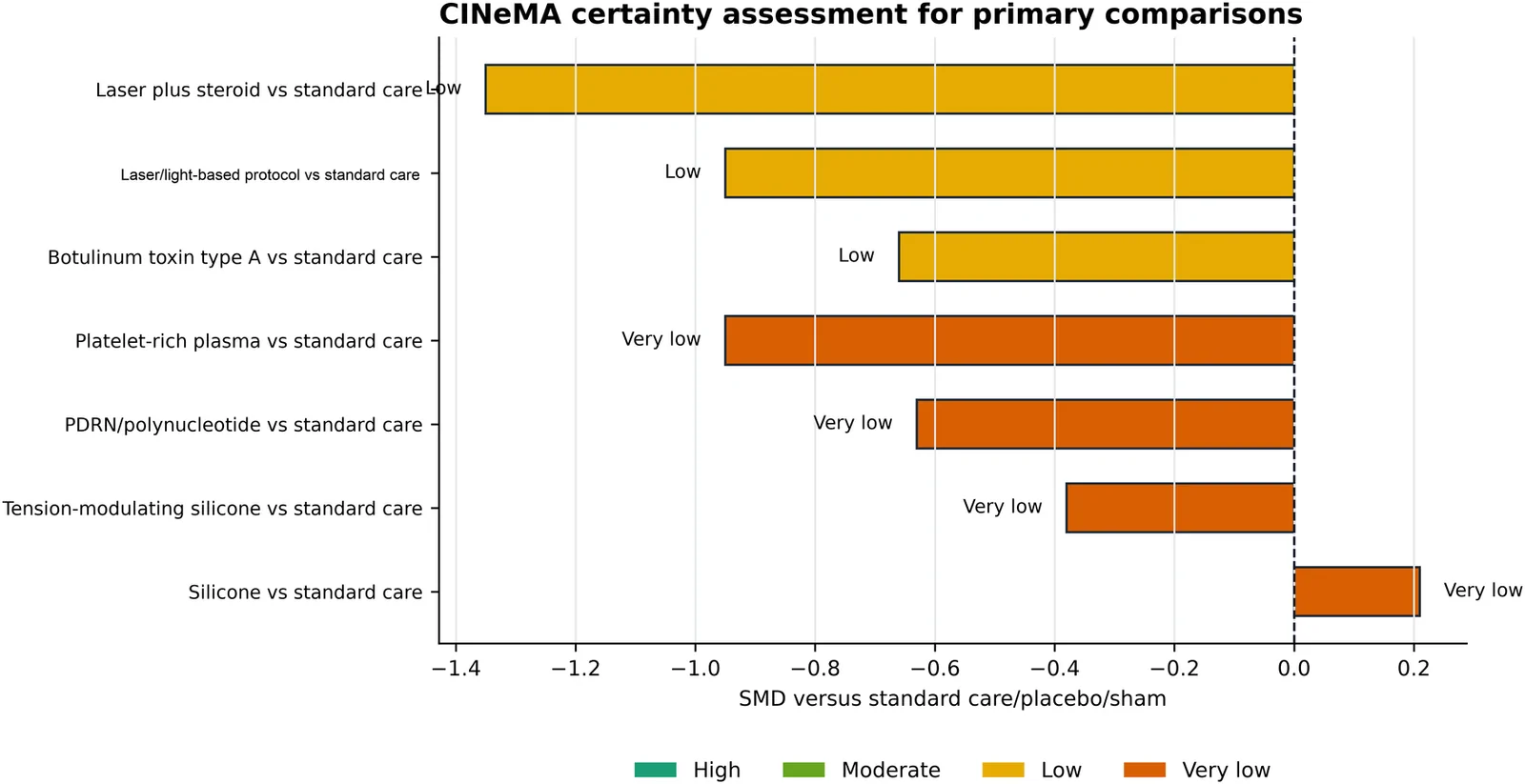
CINeMA certainty summary.

Primary network meta-analysis estimates are summarized in Table 3 and presented graphically in Figure 4; CINeMA certainty ratings for these primary comparisons are summarized in Figure 5. Unless otherwise specified, the estimates reported in the following are random-effects network estimates versus standard care/placebo/sham. Laser plus steroid showed a favorable low-certainty network estimate (SMD −1.35, 95% CI −1.87 to −0.83), and laser/light-based protocol also showed a favorable low-certainty estimate (SMD −0.95, 95% CI −1.40 to −0.50). PRP showed a favorable but very-low-certainty estimate (SMD −0.95, 95% CI −1.73 to −0.16) based on a single small study with methodological concerns. Botulinum toxin type A showed a favorable low-certainty estimate (SMD −0.66, 95% CI −1.18 to −0.14). PDRN/PN showed an imprecise very-low-certainty estimate (SMD −0.63, 95% CI −1.30 to 0.04). Silicone dressing alone did not show a clear favorable estimate (SMD 0.21, 95% CI −0.45 to 0.87), and tension-modulating silicone had an imprecise estimate (SMD −0.38, 95% CI −1.19 to 0.42). No cross-scale minimal clinically important difference (MCID) was assumed; these estimates should be interpreted using approximate standardized magnitudes and clinical context rather than as directly interchangeable scar score units.

**Table 3:** Primary network meta-analysis estimates.

Treatment	Network SMD vs. standard care/placebo/sham (95% CI)	CINeMA certainty	Approximate magnitude	Interpretation
Laser plus intralesional steroid protocol	−1.35 (−1.87 to −0.83)	Low	Large standardized difference	Favorable point estimate, but certainty is low and mechanistic synergy is not directly tested
Laser/light-based protocol	−0.95 (−1.40 to −0.50)	Low	Large standardized difference	Favorable point estimate for scar appearance; comparative hierarchy remains uncertain
Platelet-rich plasma injection	−0.95 (−1.73 to −0.16)	Very low	Large standardized difference	Favorable single-study estimate; interpretation is exploratory because evidence is very uncertain
Botulinum toxin type A	−0.66 (−1.18 to −0.14)	Low	Moderate standardized difference	Favorable point estimate; evidence remains sparse
PDRN/PN injection	−0.63 (−1.30 to 0.04)	Very low	Moderate standardized difference	Imprecise estimate crossing the null; hypothesis-generating only
Elastic/tension-modulating silicone	−0.38 (−1.19 to 0.42)	Very low	Small-to-moderate standardized difference	Imprecise estimate; may remain relevant when adherence and tension modulation are clinically feasible
Silicone dressing/sheet	0.21 (−0.45 to 0.87)	Very low	Small standardized difference	No clear favorable isolated effect in this sparse network; this should not be interpreted against silicone as low-risk foundational background scar care

### 3.6 Direct evidence structure and split-scar sensitivity

The full league matrix with CINeMA certainty ratings for comparisons versus standard care/placebo/sham is provided in Supplementary Table S7. Treatment-ordering metrics were not used to support superiority claims because the network was underpowered for reliable comparative hierarchy. Split-scar paired-data sensitivity analyses were performed using assumed within-participant correlations of *r* = 0.25, *r* = 0.50, and *r* = 0.75. One split-scar comparison was adjusted. The assumed correlation affected the precision of the silicone tension modulation comparison but did not materially change the direction of the network estimates. For tension-modulating silicone versus standard care/placebo, the estimate remained −0.38 across scenarios, with 95% CIs of −1.17 to 0.40, −1.15 to 0.38, and −1.13 to 0.36 for *r* = 0.25, *r* = 0.50, and *r* = 0.75, respectively. A dedicated PRP-exclusion sensitivity analysis was also performed because the PRP node was supported by one small RCT with methodological concerns. Excluding Pk and Chaudhary removed the PRP node and yielded a five-trial, seven-node connected network (23). Non-PRP network estimates were unchanged to two decimal places, and heterogeneity was unchanged (*τ*^2^ = 0.0216; *I*^2^ = 22.9%). These results indicate that the PRP estimate itself is single-study and exploratory, but that this study did not drive the non-PRP treatment estimates. Detailed results are provided in Supplementary Table S8.

### 3.7 Heterogeneity assessment

Network statistical heterogeneity was low in the primary connected scar-directed model. The random-effects variance was *τ*^2^ = 0.0216, corresponding to *τ* = 0.1471, and the network *I*^2^ was 22.9%. However, the low *I*^2^ value should not be read as evidence that clinical heterogeneity was absent. The primary outcome was harmonized using SMDs across POSAS, PSAS/OSAS, VSS/mVSS, SBSES, and MSS, but these instruments do not measure identical scar constructs. POSAS incorporates patient and observer scar perception; VSS/mVSS emphasizes objective scar features such as vascularity, pigmentation, pliability, and height; and SBSES/MSS place greater emphasis on cosmetic wound appearance. Differences in scale construct, assessor perspective, scar domain, follow-up window, and trial design may therefore introduce clinical heterogeneity and indirectness that are not measured by *I*^2^. For this reason, the low statistical heterogeneity estimate was interpreted cautiously and was not used to dismiss concerns about transitivity or certainty.

### 3.8 Inconsistency and small-study effects

Global and local inconsistency assessments were interpreted cautiously. Design-by-treatment and node-splitting tests did not identify statistically significant inconsistency, but the network contained few closed loops and most comparisons were informed by single studies. Therefore, non-significant inconsistency tests should not be read as proof of consistency; the network was too sparse to detect inconsistency reliably.

Publication bias and small-study effects could not be reliably assessed because the primary connected network included only six RCTs and most comparisons were informed by one study. At this evidence size, any comparison-adjusted funnel plot or asymmetry-based assessment would be exploratory, underpowered, and potentially misleading. The comparison-adjusted funnel plot was therefore removed from the main manuscript, and no inference was made regarding the presence or absence of missing unpublished trials.

### 3.9 CINeMA certainty assessment

The CINeMA certainty assessment for primary comparisons is summarized in Table 4. CINeMA assessment was performed across six domains: within-study bias, reporting bias, indirectness, imprecision, heterogeneity, and incoherence. Certainty was low or very low for all primary comparisons, driven mainly by sparse evidence, imprecision, scale and timing indirectness, and limited replication. The PRP study was judged as having some concerns rather than high risk of bias because the judgment was based on trial design and conduct, not PubMed indexing status. The study reported randomization, a concurrent control group, thyroidectomy scars, and blinded scar assessment, but protocol and outcome-reporting safeguards were incompletely reported. CINeMA certainty for PRP remained very low.

**Table 4:** CINeMA certainty assessment for primary comparisons.

Comparison	SMD (95% CI)	Bias	Indirectness	Imprecision	Heterogeneity	Incoherence	Certainty
Laser plus steroid vs. standard care	−1.35 (−1.87 to −0.83)	Some concerns	Some concerns	Some concerns	No major concerns	Some concerns	Low
Laser/light-based protocol vs. standard care	−0.95 (−1.40 to −0.50)	Some concerns	Some concerns	Some concerns	No major concerns	Some concerns	Low
Platelet-rich plasma vs. standard care	−0.95 (−1.73 to −0.16)	Some concerns	Some concerns	Major concerns	No major concerns	No major concerns	Very low
Botulinum toxin type A vs. standard care	−0.66 (−1.18 to −0.14)	Some concerns	Some concerns	Some concerns	No major concerns	Some concerns	Low
PDRN/polynucleotide vs. standard care	−0.63 (−1.30 to 0.04)	Some concerns	Some concerns	Major concerns	No major concerns	No major concerns	Very low
Tension-modulating silicone vs. standard care	−0.38 (−1.19 to 0.42)	Some concerns	Some concerns	Major concerns	No major concerns	Some concerns	Very low
Silicone vs. standard care	0.21 (−0.45 to 0.87)	Some concerns	Some concerns	Major concerns	No major concerns	Some concerns	Very low

### 3.10 Safety and treatment burden

Safety, cost, access, patient preference, and treatment burden reporting were inconsistent across included trials, so comparative safety meta-analysis was not appropriate. Available adverse-event information was extracted at the trial and intervention-class levels and is summarized in Supplementary Tables S5, S5A, and S5B. Across the available reports, no consistent serious adverse-event signal was identified, but event definitions, counts, pain scores, downtime, and patient preference outcomes were not reported uniformly. Laser/light-based interventions may involve erythema, discomfort, crusting, pigmentary change, and photoprotection needs; intralesional steroid adds potential risks of atrophy, telangiectasia, or hypopigmentation; injectable biologics add injection and preparation burden; and silicone-based strategies are comparatively low risk but adherence-dependent.

Detailed safety, cost, access, patient preference, and treatment burden extractions are provided in Supplementary Tables S5A and S5B.

### 3.11 Closure method evidence map

Closure method trials were not pooled in the primary NMA and were moved to the Supplementary Material as a descriptive evidence map (Supplementary Table S6) (24). These studies address intraoperative wound-edge approximation, closure materials, track marks, operative time, early wound support, or surgeon preference, whereas the primary network addresses postoperative modulation of inflammation, collagen remodeling, pigmentation, erythema, and tension. Mixing closure techniques with scar-directed therapies would weaken transitivity and reduce the interpretability of the main clinical question.

## 4 Discussion

This network meta-analysis should be interpreted as a cautious comparative synthesis of a small evidence base rather than as a definitive treatment hierarchy. Laser plus steroid and laser/light-based protocols showed favorable low-certainty point estimates, while PRP, botulinum toxin type A, and PDRN/PN remained clinically interesting but uncertain options. Because the network contained only six RCTs and most nodes were informed by single studies, certainty was low or very low, and comparative ordering remains unstable.

The main methodological gain of this review is the separation of two clinical questions that are often conflated in scar literature. Closure methods address wound-edge approximation during surgery, whereas scar-directed interventions attempt to modulate postoperative wound healing, inflammation, collagen remodeling, pigmentation, vascularity, and tension. Accordingly, closure method findings are not interpreted as part of the scar-directed treatment effects in this Discussion; they are retained only as a supplementary evidence map to clarify the boundary between operative technique and postoperative scar modulation.

### 4.1 Interpreting favorable laser-containing estimates

The favorable point estimates for laser-containing protocols are biologically plausible, but the included trials did not directly test mechanistic synergy between laser treatment and steroid injection. Mechanistic interpretations in this section are intended to contextualize observed estimates and should be considered hypothesis-generating rather than findings of the present NMA. Fractional carbon dioxide laser and non-ablative laser may remodel collagen architecture and improve texture, while light- or vascular-targeted modalities may reduce erythema and visible vascularity (25–27).

Intralesional steroid may suppress fibroblast activity and excessive collagen deposition, providing a plausible rationale for combining energy-based treatment with anti-inflammatory modulation in early thickened scars. However, the present evidence does not establish that these mechanisms act synergistically in thyroidectomy scars; it only demonstrates favorable low-certainty point estimates for laser-containing protocols.

### 4.2 Clinical considerations rather than recommendations

The clinical implications of this work should be framed as considerations for shared decision-making and future trials, not as guidelines. For all patients, meticulous wound handling, tension reduction, sun protection, realistic counseling, and basic topical or mechanical scar support remain foundational. Silicone products should therefore not be dismissed because silicone dressing alone did not show a favorable isolated network estimate in this sparse anterior neck network. Cochrane reviews and international scar management recommendations support silicone gel or sheeting as a first-line non-invasive scar prevention and scar management option, particularly because it is low risk, accessible, and compatible with background wound care (2, 3, 5, 28, 29). The present NMA does not overturn that role; rather, it suggests that silicone should be interpreted primarily as foundational background care rather than as a stand-alone procedural node competing with laser or injectable interventions.

The apparently unfavorable isolated silicone estimate should not be interpreted as evidence against silicone-based scar prevention. Silicone products remain widely recommended as first-line basic scar care because they are non-invasive, low risk, accessible, and compatible with broader wound support measures. Several factors may explain the discrepancy observed in this anterior neck NMA. First, thyroidectomy scars lie in a mobile, sometimes high-tension anatomical region affected by swallowing, neck extension, rotation, and platysmal movement, which may reduce adherence and make delivered tension modulation difficult to standardize. Second, silicone protocols differed across trials in product type, start time, daily wear duration, total treatment duration, adherence monitoring, and cointerventions such as taping, massage advice, sun protection, or routine scar education. Third, follow-up ranged from 3 to 12 months, spanning different phases of erythema, pigmentation, pliability, and scar maturation. Fourth, the silicone evidence base was small and included split-scar or active-comparator designs, leaving the isolated network estimate vulnerable to imprecision and ceiling effects in clean thyroidectomy incisions. Therefore, silicone should be interpreted as foundational background scar care, whereas the present analysis estimates its isolated incremental effect within a sparse connected network rather than its full role in multimodal scar prevention.

Botulinum toxin type A may be a reasonable research target for high-tension anterior neck wounds where dynamic muscle pull could contribute to scar widening or hypertrophy, but the current evidence is not strong enough to support routine use (30, 31). PRP and PDRN/PN should likewise be discussed, if at all, as investigational or hypothesis-generating options because evidence is sparse, certainty is very low, and cost, access, preparation variability, and treatment burden may limit routine thyroid surgery implementation. In the PRP-exclusion sensitivity analysis, PRP could no longer be estimated because the node was supported only by the excluded study; however, the remaining non-PRP network estimates were unchanged. This supports treating PRP as a separate, very-low-certainty signal rather than as a driver of the overall treatment structure.

At later early follow-up, persistent erythema, pigmentation, texture irregularity, or early thickening may justify referral for scar assessment and possible energy-based treatment in selected patients. These statements should be understood as clinical considerations generated from low-certainty evidence, not as directives. Treatment decisions should incorporate patient preference, pigmentary risk, downtime, cost, local expertise, and willingness to undergo repeated visits.

### 4.3 Safety, cost-effectiveness, and real-world feasibility of regenerative injectables

The real-world value of PRP and PDRN/PN depends on more than scar score direction. Neither the PRP nor PDRN/PN evidence base provided formal cost-effectiveness data, and both interventions introduce resource requirements that are not captured by SMDs. PRP requires blood collection, preparation or centrifugation, staff time, sterile handling, injection, and protocol standardization (32, 33). PDRN/PN depends on product sourcing, local regulatory approval, formulation, dosing schedule, and reimbursement conditions (34, 35). In routine thyroid surgery, where the target outcome is often aesthetic scar prevention rather than treatment of a disabling complication, these costs must be weighed against low or very low certainty, small-trial evidence, and uncertain durability.

For this reason, regenerative injectables should be framed as investigational adjuncts rather than routine first-line scar prevention strategies. Future trials should prospectively collect direct costs, procedure time, number of visits, pain, downtime, patient willingness to repeat treatment, and preference-sensitive outcomes. Such data are particularly important for thyroidectomy practice, where scalable interventions must fit endocrine surgery workflows and remain acceptable to patients who may already have access to lower-burden background care such as silicone, taping, sun protection, and scar education.

### 4.4 Comparison with previous systematic reviews

Previous reviews of thyroidectomy wound closure have mainly addressed tissue adhesive, staples, strips, and subcuticular or intradermal sutures. Those studies are clinically useful for operative technique, but they answer a different question from postoperative scar modulation. Broader scar management reviews support silicone products as foundational first-line care and discuss botulinum toxin, lasers, steroid injection, PRP, and regenerative products across heterogeneous scar types. The present review adds a focused anterior neck scar-directed framework but does not overturn established low-risk background care.

The discrepancy between guideline support for silicone and the low isolated silicone estimate in this network should be interpreted cautiously. Silicone-related evidence in this anterior neck network was limited, application protocols differed, and standard care may have included scar advice or wound support that reduced incremental contrast. Thus, silicone remains clinically valuable as accessible background scar prevention, even if it did not appear favorable as an isolated comparative node in this sparse model.

### 4.5 Methodological considerations and future trials

The primary network showed low statistical heterogeneity and no statistically significant global inconsistency, but these findings should not be overinterpreted. Non-significant node-splitting and design-by-treatment results do not prove consistency; they indicate only that no inconsistency was detectable with the available evidence. Sparse networks may lack power to detect inconsistency, and low heterogeneity does not remove imprecision, indirectness, or within-study bias.

Future trials should standardize background care, use POSAS or comparable patient-and-observer scar scales, prespecify the primary time point, report complete means and standard deviations, and capture pigmentary events, pain, downtime, treatment burden, cost, and patient willingness to repeat treatment. Split-scar or split-incision designs should report paired statistics so that precision is not distorted by arm-level analysis. Trials should also stratify clinically meaningful risk, including prior hypertrophic scarring, Fitzpatrick phototype, incision length, closure tension, early erythema, early widening, and early thickening.

### 4.6 Limitations

Several limitations should temper interpretation. The primary connected network was small, with six randomized trials and limited closed loops. Although network *I*^2^ was 22.9%, this statistical summary does not capture all clinically relevant heterogeneity. Scar scales differed in construct, with POSAS emphasizing patient and observer scar perception, VSS/mVSS emphasizing objective scar characteristics, and SBSES/MSS emphasizing aesthetic wound appearance. No universal MCID could be applied across these scales; the SMD should therefore be interpreted as a standardized direction and approximate magnitude of scar quality difference rather than a directly interchangeable clinical unit. Similarly, 3- to 12-month outcomes span early and intermediate scar maturation, contributing to indirectness. Split-scar and split-incision designs required paired-data assumptions where paired standard deviations were unavailable; sensitivity analyses suggested that these assumptions mainly affected precision. Publication bias and small-study effects could not be reliably assessed because the primary network contained only six RCTs, with most treatment comparisons informed by single studies. Accordingly, the absence of a reliable funnel plot assessment should not be interpreted as evidence for or against missing unpublished trials. Adverse events, treatment burden, cost, and patient preference were not consistently reported across intervention classes.

### 4.7 Conclusions

A focused primary network of scar-directed interventions provides a more defensible synthesis than a broad mixed network of scar therapies and closure techniques. In this sparse network of randomized trials, laser plus steroid and laser/light-based protocols showed favorable low-certainty point estimates compared with standard care/placebo. PRP, botulinum toxin type A, and PDRN/PN remain clinically interesting but uncertain options, whereas silicone-based strategies should continue to be considered foundational background scar care rather than judged solely by their isolated network estimate. Because certainty was low or very low across comparisons and the network was underpowered for reliable comparative hierarchy, these findings should be interpreted as hypothesis-generating and as a framework for individualized scar prevention research rather than as a definitive treatment hierarchy.

## Data Availability

The original contributions presented in the study are included in the article/Supplementary Material, further inquiries can be directed to the corresponding author.
