# Supplementary material for: Early scar-directed interventions for thyroidectomy scars: a systematic review and network meta-analysis
Source: Front Surg. 2026 Sep 8;13:1914326. doi: 10.3389/fsurg.2026.1914326 (PMC13593765; doi:10.3389/fsurg.2026.1914326)
Supplement: Supplementary file 1 [file Datasheet1.pdf]

Supplementary Material

Early scar-directed interventions for thyroidectomy scars: a systematic review and network meta-analysis

This supplementary file was revised in response to Reviewer 1. It provides the complete independent database search strings, transitivity assessment, SMD clinical anchoring, split-scar paired-data sensitivity analysis, safety and treatment-burden summary, closure-method evidence map, and league table with CINeMA certainty ratings.

Supplementary Table S1. Complete independent database search strategies

Database	Search window	Search string
PubMed/MEDLINE	Database inception to June 17, 2026	((thyroidectomy[Title/Abstract] OR parathyroidectomy[Title/Abstract] OR cervicotomy[Title/Abstract] OR "anterior neck"[Title/Abstract] OR "neck incision"[Title/Abstract]) AND (scar[Title/Abstract] OR scars[Title/Abstract] OR scarring[Title/Abstract] OR cicatrix[Title/Abstract] OR cosmesis[Title/Abstract] OR cosmetic[Title/Abstract] OR POSAS[Title/Abstract] OR "Patient and Observer Scar Assessment Scale"[Title/Abstract] OR Vancouver[Title/Abstract] OR VSS[Title/Abstract] OR "Stony Brook"[Title/Abstract] OR SBSES[Title/Abstract] OR "Manchester Scar Scale"[Title/Abstract]) AND (botulinum[Title/Abstract] OR Botox[Title/Abstract] OR laser[Title/Abstract] OR light[Title/Abstract] OR photobiomodulation[Title/Abstract] OR silicone[Title/Abstract] OR polynucleotide[Title/Abstract] OR PDRN[Title/Abstract] OR "platelet-rich

		plasma"[Title/Abstract] OR PRP[Title/Abstract] OR "wound protector"[Title/Abstract] OR "tissue adhesive"[Title/Abstract] OR suture[Title/Abstract] OR staples[Title/Abstract]) AND (randomized[Title/Abstract] OR randomised[Title/Abstract] OR randomly[Title/Abstract] OR trial[Title/Abstract] OR "controlled trial"[Title/Abstract]))
Embase	Database inception to June 17, 2026	('thyroidectomy':ti,ab OR 'parathyroidectomy':ti,ab OR 'cervicotomy':ti,ab OR 'anterior neck':ti,ab OR 'neck incision':ti,ab) AND ('scar':ti,ab OR 'scars':ti,ab OR 'scarring':ti,ab OR 'cicatrix':ti,ab OR 'cosmesis':ti,ab OR 'cosmetic':ti,ab OR 'posas':ti,ab OR 'patient and observer scar assessment scale':ti,ab OR 'vancouver scar scale':ti,ab OR 'vss':ti,ab OR 'stony brook scar evaluation scale':ti,ab OR 'sbses':ti,ab OR 'manchester scar scale':ti,ab) AND ('botulinum toxin':ti,ab OR 'botox':ti,ab OR 'laser':ti,ab OR 'light therapy':ti,ab OR 'photobiomodulation':ti,ab OR 'silicone':ti,ab OR 'polynucleotide':ti,ab OR 'polydeoxyribonucleotide':ti,ab OR 'pdrn':ti,ab OR 'platelet rich plasma':ti,ab OR 'prp':ti,ab OR 'wound protector':ti,ab OR 'tissue adhesive':ti,ab OR 'suture':ti,ab OR 'staple':ti,ab) AND ('randomized controlled trial'/exp OR random*:ti,ab OR randomised:ti,ab OR randomized:ti,ab OR trial:ti,ab)
Cochrane CENTRAL	Database inception to June 17, 2026	(thyroidectomy OR parathyroidectomy OR cervicotomy OR "anterior neck" OR "neck incision") AND (scar OR scars OR scarring OR cicatrix OR cosmesis OR cosmetic OR POSAS OR

		"Patient and Observer Scar Assessment Scale" OR "Vancouver Scar Scale" OR VSS OR "Stony Brook Scar Evaluation Scale" OR SBSES OR "Manchester Scar Scale") AND (botulinum OR Botox OR laser OR light OR photobiomodulation OR silicone OR polynucleotide OR polydeoxyribonucleotide OR PDRN OR "platelet-rich plasma" OR PRP OR "wound protector" OR "tissue adhesive" OR suture OR staples)

Note: PubMed/MEDLINE, Embase, and CENTRAL were searched independently. Reference tracking, DOI/Crossref verification, ClinicalTrials.gov, WHO ICTRP, PMC/open full-text retrieval, and manual checking were used as supplementary verification sources.

Supplementary Table S2. Transitivity assessment for the primary scar-directed network

Potential effect modifier	Observed distribution	Transitivity judgment
Surgical site and incision	Included primary-network trials evaluated thyroidectomy or anterior-neck linear surgical scars.	Supports transitivity for anterior neck scar prevention, but limits extrapolation to non-neck scars.
Scar phenotype	The network addressed early postoperative linear scars rather than mature keloids, burns, traumatic scars, or acne scars.	Supports pooling within a prevention-oriented surgical-scar framework.
Intervention timing	Interventions were intended to modulate early wound healing, inflammation, collagen remodeling, pigmentation, erythema, or tension.	Mechanisms were sufficiently aligned for a scar-directed network; closure techniques were excluded from the primary model.
Outcome scale	POSAS, PSAS/OSAS, VSS/mVSS, SBSES, and MSS were used.	All scales assess scar quality/cosmesis, but domains differ; therefore SMD was used and certainty was downgraded for indirectness where

		appropriate.
Follow-up window	Primary outcomes were selected at the longest available 3- to 12-month follow-up.	This window captures early-to-intermediate scar maturation; residual timing heterogeneity was acknowledged as indirectness.
Comparator context	Comparators included standard care, placebo, sham, silicone, and active scar-directed bridge comparisons.	Generally acceptable for a connected scar-directed network, but sparse single-study nodes limit confidence.
Trial design	Parallel, multi-arm, and split-scar/split-incision trials were included.	Split-scar paired-data assumptions were handled through correlation-based sensitivity analyses.
Population	Most trials were small, single-center, and predominantly Asian cohorts.	Clinical generalizability and effect-modifier balance remain limited.

Supplementary Table S3. Clinical anchoring of standardized mean differences

SMD magnitude	Clinical framing	Interpretation used in this revision
Approximately 0.2 SD	Small standardized difference	May reflect a subtle scar-score difference that may not be readily visible or clinically important in an individual patient.
Approximately 0.5 SD	Moderate standardized difference	May correspond to a visible or patient-noticeable difference depending on the scale domain and baseline scar severity.
Approximately 0.8 SD or larger	Large standardized difference	Suggests a potentially meaningful scar-quality difference, but interpretation remains scale-dependent.

No universal MCID	Not available across all included scar scales	Because POSAS, VSS/mVSS, SBSES, PSAS/OSAS, and MSS capture different constructs, cross-scale MCID conversion was not assumed.

Supplementary Table S4. Split-scar paired-data sensitivity analysis

treatment	r=0.25	r=0.50	r=0.75
BTA	-0.66 (-1.18 to -0.14)	-0.66 (-1.18 to -0.14)	-0.66 (-1.18 to -0.14)
LASER_LIGHT	-0.95 (-1.40 to -0.50)	-0.95 (-1.40 to -0.50)	-0.95 (-1.40 to -0.50)
LASER_STEROID	-1.35 (-1.87 to -0.83)	-1.35 (-1.87 to -0.83)	-1.35 (-1.87 to -0.83)
PDRN_PN	-0.63 (-1.30 to 0.04)	-0.63 (-1.30 to 0.04)	-0.63 (-1.30 to 0.04)
PRP	-0.95 (-1.73 to -0.16)	-0.95 (-1.73 to -0.16)	-0.95 (-1.73 to -0.16)
SILICONE	0.21 (-0.45 to 0.87)	0.21 (-0.45 to 0.87)	0.21 (-0.45 to 0.87)
SILICONE_TENSION	-0.38 (-1.17 to 0.40)	-0.38 (-1.15 to 0.38)	-0.38 (-1.13 to 0.36)

Note: One split-scar comparison contributed to the primary connected network. The primary analysis used an assumed within-participant correlation of r = 0.50; sensitivity analyses used r = 0.25 and r = 0.75. The assumed correlation mainly affected precision for the tension-modulating silicone comparison and did not materially change the direction of network estimates.

Supplementary Table S5. Safety, treatment burden, and access considerations

Intervention class	Current NMA efficacy signal	CINeMA certainty	Available safety/adverse-	Treatment burden and session considerations	Cost/access considerations	Clinical interpretation

			event information			
Laser/light-based protocol	Favorable point estimate versus standard care/placebo (SMD -0.95, 95% CI -1.40 to -0.50)	Low	Adverse-event reporting was limited. Expected or reported burdens include transient erythema, discomfort, crusting, edema, pigmentary change, and need for post-procedure photoprotection; serious events were not consistently reported.	Requires device-based procedural care; often delivered across repeated sessions or protocolized postoperative visits; requires laser/light expertise and follow-up.	Higher resource requirement than silicone or simple wound care; availability depends on access to dermatology/plastic-surgery laser services.	May be considered for persistent erythema, color mismatch, vascularity, pigmentation, or texture concerns, but comparative superiority remains uncertain.
Laser plus intralesional steroid protocol	Favorable point estimate versus standard care/placebo (SMD -1.35, 95% CI -1.87 to -0.83)	Low	Safety reporting was sparse. Potential burdens include injection pain, bruising, transient erythema or crusting after laser, and steroid-related atrophy, telangiectasia, or hypopigmentation; these events were not uniformly	Requires combined procedural expertise, laser access, and intralesional injection; may require repeated or staged visits depending on scar evolution.	Higher procedural cost and operator dependence; access may be limited outside specialized scar or laser clinics.	Biologically plausible for early thickening or hypertrophic tendency, but evidence remains low certainty and mechanism-based interpretation should be treated as hypothesis-generating.

			captured.			
Botulinum toxin type A	Favorable point estimate versus standard care/placebo (SMD -0.66, 95% CI -1.18 to -0.14)	Low	Reported serious adverse events were uncommon or not emphasized in available reports. Potential burdens include injection pain, bruising, and transient local weakness or functional concern, particularly in the mobile anterior neck.	Usually involves intraoperative or early postoperative injection; lower visit burden than repeated laser protocols, but requires correct dose, placement, and anatomical expertise.	Moderate-to-high product cost; access and reimbursement vary; operator training influences feasibility.	May be most relevant when dynamic neck tension or early widening is the dominant concern; current evidence supports cautious consideration rather than routine use.
Platelet-rich plasma	Favorable point estimate versus standard care/placebo (SMD -0.95, 95% CI -1.73 to -0.16)	Very low	Safety reporting was limited in a single small study. Potential burdens include blood draw, injection discomfort, bruising, swelling, and preparation variability; autologous preparation may reduce immunologic risk but does not remove procedural	Requires blood collection, centrifugation/preparation, and injection; adds operative or procedural time; protocols are not standardized.	Cost depends on preparation system, staffing, and local availability; standardization and quality control are barriers to routine implementation.	Exploratory signal only. The estimate is supported by one small study with methodological concerns and very low certainty; sensitivity excluding this study should be reported separately.

			burden.			
PDRN/polynucleotide injection	Imprecise favorable point estimate versus standard care/placebo (SMD -0.63, 95% CI -1.30 to 0.04)	Very low	Adverse-event reporting was sparse. Potential burdens include injection pain, bruising, swelling, erythema, and uncertainty around product-specific reactions; serious adverse events were not consistently captured.	Typically requires injectable treatment and may require repeated administrations; dose, schedule, and product formulation vary.	Cost and availability vary substantially by region; product regulation, sourcing, and reimbursement may limit generalizability.	Biologically plausible regenerative adjunct, but the confidence interval crosses the null and certainty is very low.
Silicone dressing/sheet	No clear favorable isolated estimate versus standard care/placebo in the network (SMD 0.21, 95% CI -0.45 to 0.87)	Very low	Generally low procedural risk. Main burdens include local irritation, itching, maceration, poor adherence, discomfort with prolonged wear, and reduced compliance.	Requires prolonged daily wear and patient adherence; effectiveness depends heavily on correct application and duration of use.	Relatively accessible and lower cost than procedural or injectable therapies, though cumulative cost and adherence burden may matter.	Should not be dismissed because of its isolated network estimate. Silicone may function best as foundational background scar care rather than as a standalone active node.
Tension-modulating silicone	Imprecise favorable point estimate versus standard care/placebo	Very low	Safety profile is similar to silicone sheeting, with possible irritation, itching, discomfort,	Requires correct placement and sustained wear to reduce tension; compliance and neck mobility may affect	Moderate cost and widely variable product availability; less resource-intensive than	May be relevant for high-tension or widening-prone anterior-neck scars, but the

	(SMD -0.38, 95% CI -1.19 to 0.42)		maceration, and adherence problems; adverse-event reporting was limited.	delivered benefit.	procedural interventions but more adherence-dependent.	estimate is indirect and imprecise.

Supplementary Table S6. Closure-method evidence map retained outside the primary NMA

Study	Year	PMID	DOI	Population	Closure comparison	N	Scar/cosmetic outcomes	Follow-up	Reason not pooled in primary NMA
O'Leary et al.	2013	23261023	10.1016/j.surg.2012.08.063	Thyroidectomy/parathyroidectomy	Adhesive strips vs absorbable subcutaneous sutures	82	Hollander cosmesis; Likert cosmetic assessment; pain; complications	6 weeks	Intraoperative/early closure method; different mechanism and decision context from postoperative scar-directed therapy; retained as descriptive closure evidence only.
Teoh et al.	2019	30446424	10.1016/j.asjsur.2018.09.014	Thyroidectomy	Tissue adhesive vs subcuticular suture	96	SBSES at 6 weeks; POSAS observer	6 weeks and 3 months	Closure-material comparison; does not

							component at 3 months		evaluate postoperative scar modulation; retained descriptively.
Lombardi et al.	2011	22229233	Not reported in available inventory	Thyroidectomy	Four-arm comparison of intradermal suture variants, staples, and tissue adhesive	140	Modified Vancouver Scar Scale; PSAS	3 months	Multi-arm closure-technique study; clinically and mechanistically distinct from scar-directed interventions; retained descriptively.
Selvadurai et al.	1997	9244079	Not reported in available inventory	Thyroid and parathyroid surgery	Subcuticular suture vs metal clips	80	Patient, surgeon, and observer cosmetic scales; pain	Not fully extractable from inventory	Closure-technique comparison; retained descriptively because complete harmonized scar-outcome data were not used in the primary NMA.
Guarino et	201	248594	10.1016/j.ijsu.2014.05.	Cervicotomy incisions	Staples vs subcuticular	20	Cosmesis and	Not fully extracta	Small closure-method study

al.	4	05	018		closure		closure outcomes	ble from inventory	with sparse extractable information; retained as descriptive evidence map only.
Pronio et al.	2011	No PMID identified	10.1007/s00238-010-0477-6	Thyroid surgery	Octyl-2-cyanoacrylate adhesive vs metal clips	70	Cosmesis; satisfaction; complications	Not fully extractable from inventory	Closure-method comparison; retained descriptively rather than quantitatively synthesized with scar-directed interventions.
Reed/Lennard	1997	9043474	10.1002/bjs.1800840142	Neck wound closure	Clips vs subcuticular polydioxanone	Not reported in abstract	No abstract-level scar outcome available in inventory	Not fully extractable from inventory	Closure-method record; retained descriptively pending complete extractable outcome data.
Maw et al.	1997	9055170	Not reported in available inventory	Head and neck incisions	Octylcyanoacrylate tissue adhesive vs suture	50	Validated wound scale; blinded photographic visual analogue	4-6 weeks	Broader head-and-neck closure study; not thyroidectomy-specific scar-directed

							scale		therapy; retained descriptively.

Note: These studies address wound-edge approximation or closure materials at the time of operation. They were not pooled with postoperative scar-directed interventions because the clinical question, mechanism, timing, and decision context differ.

Supplementary Table S7. League table with CINeMA certainty ratings

Comparison	Network SMD	95% CI	CINeMA certainty	Interpretation
Laser plus steroid vs standard care	-1.35	-1.87 to -0.83	Low	Favorable estimate but based on sparse direct and indirect evidence from a small connected network.
Laser/light-based protocol vs standard care	-0.95	-1.40 to -0.50	Low	Favorable estimate supported by direct bridge evidence but limited by sparse loops and outcome-scale heterogeneity.
Platelet-rich plasma vs standard care	-0.95	-1.73 to -0.16	Very low	Favorable single-study estimate; certainty limited by small sample size, imprecision, incomplete reporting of protocol/outcome-reporting safeguards, and reliance on one study. Certainty remains very low despite revision of the RoB 2 overall judgment

				to some concerns.
Botulinum toxin type A vs standard care	-0.66	-1.18 to -0.14	Low	Favorable estimate with direct evidence and indirect bridge evidence; certainty limited by network sparsity.
PDRN/polynucleotide vs standard care	-0.63	-1.30 to 0.04	Very low	Direction favors PDRN/PN but confidence interval crosses the null and evidence is sparse.
Tension-modulating silicone vs standard care	-0.38	-1.19 to 0.42	Very low	Estimate is imprecise and indirect because direct evidence compares against silicone rather than standard care.
Silicone vs standard care	0.21	-0.45 to 0.87	Very low	No favorable estimate in the current network; evidence is indirect and imprecise.

Note: The CINeMA certainty ratings shown here apply to comparisons versus standard care/placebo/sham. Other treatment-to-treatment contrasts are available in the league matrix but should be interpreted descriptively because the network is sparse and most nodes are informed by single studies.

Supplementary Table S8. PRP-exclusion sensitivity analysis

Scenario	Included primary-network RCTs	Connected nodes	Direct comparisons	tau squared	I squared, %	Retained nodes

Primary network	6	8	10	0.0216	22.9	BTA; LASER_LIGHT; LASER_STEROID; PDRN_PN; PRP; SILICONE; SILICONE_TENSION; STD
PRP-excluded sensitivity	5	7	9	0.0216	22.9	BTA; LASER_LIGHT; LASER_STEROID; PDRN_PN; SILICONE; SILICONE_TENSION; STD

Treatment	Primary network estimate	PRP-excluded estimate	Change in point estimate	Interpretation
BTA	-0.66 (-1.18 to -0.14)	-0.66 (-1.18 to -0.14)	-0.00	No material change in non-PRP estimate.
LASER_LIGHT	-0.95 (-1.40 to -0.50)	-0.95 (-1.40 to -0.50)	0.00	No material change in non-PRP estimate.
LASER_STEROID	-1.35 (-1.87 to -0.83)	-1.35 (-1.87 to -0.83)	-0.00	No material change in non-PRP estimate.
PDRN_PN	-0.63 (-1.30 to 0.04)	-0.63 (-1.30 to 0.04)	-0.00	No material change in non-PRP estimate.
PRP	-0.95 (-1.73 to -0.16)	Node removed		PRP node removed because it was supported only by the excluded single study.

SILICONE	0.21 (-0.45 to 0.87)	0.21 (-0.45 to 0.87)	0.00	No material change in non-PRP estimate.
SILICONE_TENSION	-0.38 (-1.19 to 0.42)	-0.38 (-1.19 to 0.42)	0.00	No material change in non-PRP estimate.

Note: The PRP node was removed after excluding PK/Chaudhary et al. 2025 because no other PRP randomized trial contributed to the connected network. In accordance with the revised main analysis, this sensitivity analysis is interpreted using effect estimates and network geometry rather than treatment-ordering metrics.

Supplementary Figure S8. PRP-excluded sensitivity forest plot

Negative standardized mean differences favor the active scar-directed intervention. PRP is absent because its only contributing study was excluded.

Supplementary Table S5A. Trial-level adverse-event, burden, cost, and patient-preference extraction

Study/source	Intervention class	Intervention details	Reported adverse-event information	Serious adverse events	Pain/burden reporting	Patient preference reporting	Cost reporting	Interpretive limitation
Li/Zhang et al. 2026 (PMID 41882220)	Laser/light-based protocol; botulinum toxin type A; silicone control	CO2 ablative fractional laser plus intense pulsed light; BTX-A injection; silicone dressing control	The abstract states that adverse-event reports were assessed	No consistent serious adverse-event signal was available from the	Pain scores were reported as lower with BTX-A than CO2AFL-IPL in the available abstract-level verification.	Not systematically reported in the NMA extraction.	No formal cost-effectiveness analysis reported.	Safety signal is clinically useful but cannot be pooled because event definitions and counts were not consistently

			and that BTX-A had fewer adverse events and lower pain scores than CO2AFL-IPL therapy. Specific event counts were not extracted into the aggregate NMA dataset.	aggregate extraction.				available.
Chung et al. 2021 (PMID 33567135)	Laser/light-based protocol; laser plus intralesional steroid protocol	Early scar-management protocol and nonablative fractional laser plus intralesional triamcinolone protocol	Adverse-event counts were not consistently extractable from the available aggregate dataset.	Not consistently reported in extractable form.	Requires laser/light treatment visits; steroid injection adds injection-related burden.	Not systematically reported in extractable form.	No formal cost-effectiveness analysis reported.	Safety and burden must be interpreted descriptively rather than quantitatively.
Chang/Lee et al. 2020	Botulinum toxin type A	Intraoperative botulinum toxin	Serious adverse	No consistent	Single injection strategy; procedural burden lower	Not systematicall	No formal cost-	Product cost and

(PMID 32245256)		type A platysma injection versus saline/placebo	events were not emphasized in the aggregate extraction; potential injection-related effects include pain, bruising, and transient local weakness.	serious adverse-event signal available from aggregate extraction.	than repeated device-based protocols.	y reported in extractable form.	effectivenes s analysis reported.	dose/placement expertise affect real-world feasibility.
PK/Chaudhary et al. 2025 (PMID 40621269)	Platelet-rich plasma	Intraoperative autologous PRP injection before closure versus standard subcuticular closure	Safety reporting was limited in the single small PRP trial. The aggregate extraction did not provide consistently extractable adverse-	No consistent serious adverse-event signal available from aggregate extraction.	Requires blood draw, centrifugation/preparation, injection, and added operative/procedural time.	Not systematically reported in extractable form.	No formal cost-effectivenes s analysis reported.	Single-study evidence; PRP node is removed in exclusion sensitivity analysis.

			event counts.					
Kim et al. 2023 (PMID 35713247)	PDRN/PN injection	Early postoperative PDRN injections versus untreated/control care	Adverse-event reporting was sparse in the aggregate extraction.	Not consistently reported in extractable form.	Injectable product; may require repeated administrations depending on protocol.	Not systematically reported in extractable form.	No formal cost-effectiveness analysis reported.	Product availability, regulation, and pricing may differ substantially by region.
Jiang et al. 2022 (PMID 35852693)	Silicone dressing/sheet; tension-modulating silicone	Elastic silicone occlusive sheeting versus silicone occlusive sheeting	Aggregate extraction did not identify consistently extractable event counts. Expected low-risk burdens include irritation, itching, maceration, discomfort, and adherence problems.	No consistent serious adverse-event signal available from aggregate extraction.	Requires correct application and sustained wear; neck mobility may affect adherence.	Not systematically reported in extractable form.	No formal cost-effectiveness analysis reported.	Clinical value depends strongly on adherence and background care standardization.

Note: This table extracts safety and burden information available in the aggregate evidence inventory. Because adverse-event definitions, event counts, pain scores, downtime, cost, and patient-preference outcomes were inconsistently reported, quantitative safety meta-analysis was not performed.

Supplementary Table S5B. Intervention-class safety profile comparison

Intervention class	Reported or expected adverse-event profile	Treatment burden	Cost/access considerations	Clinical interpretation
Laser/light-based protocol	Transient erythema, discomfort, crusting, edema, pigmentary change, photoprotection needs	Multiple procedural visits; device access; laser expertise	Higher resource requirement than silicone/background care	Potentially useful for erythema, pigmentation, vascularity, or texture; safety reporting remains heterogeneous.
Laser plus intralesional steroid	Laser-related effects plus injection pain/bruising; steroid atrophy, telangiectasia, or hypopigmentation	Combined laser and injection expertise; staged visits may be needed	Higher procedural cost and operator dependence	Mechanistically plausible for early thickening, but adverse-event capture is incomplete.
Botulinum toxin type A	Injection pain, bruising, transient local weakness or functional concern	Single injection or limited-visit strategy; dose and placement expertise required	Product cost and reimbursement/access variability	May be feasible in selected high-tension scars but not supported for routine use.
PRP	Blood draw, injection discomfort, bruising, swelling, preparation variability	Blood collection, centrifugation/preparation, sterile handling, injection, added time	Preparation system, staffing, quality control, and standardization costs	Exploratory single-study signal; PRP node disappears after exclusion sensitivity.
PDRN/PN	Injection pain, bruising, swelling, erythema, product-specific uncertainty	Injectable product; possible repeated administrations; variable dosing schedule	Regional product availability, regulation, sourcing, pricing, reimbursement barriers	Biologically plausible but very-low-certainty and not yet routine care.
Silicone dressing/sheet	Irritation, itching, maceration,	Prolonged daily wear; patient	Lower procedural cost, but	Appropriate as foundational

	discomfort, adherence problems	adherence central	cumulative product cost and adherence burden matter	low-burden background care.
Tension-modulating silicone	Similar silicone-related irritation/maceration/adherence issues	Correct placement and sustained wear; neck mobility affects adherence	Moderate product cost; less resource-intensive than procedures	Potentially relevant when tension is the target, but evidence remains imprecise.

Supplementary Figure S9. Mechanistic separation of closure methods and early scar-directed therapies

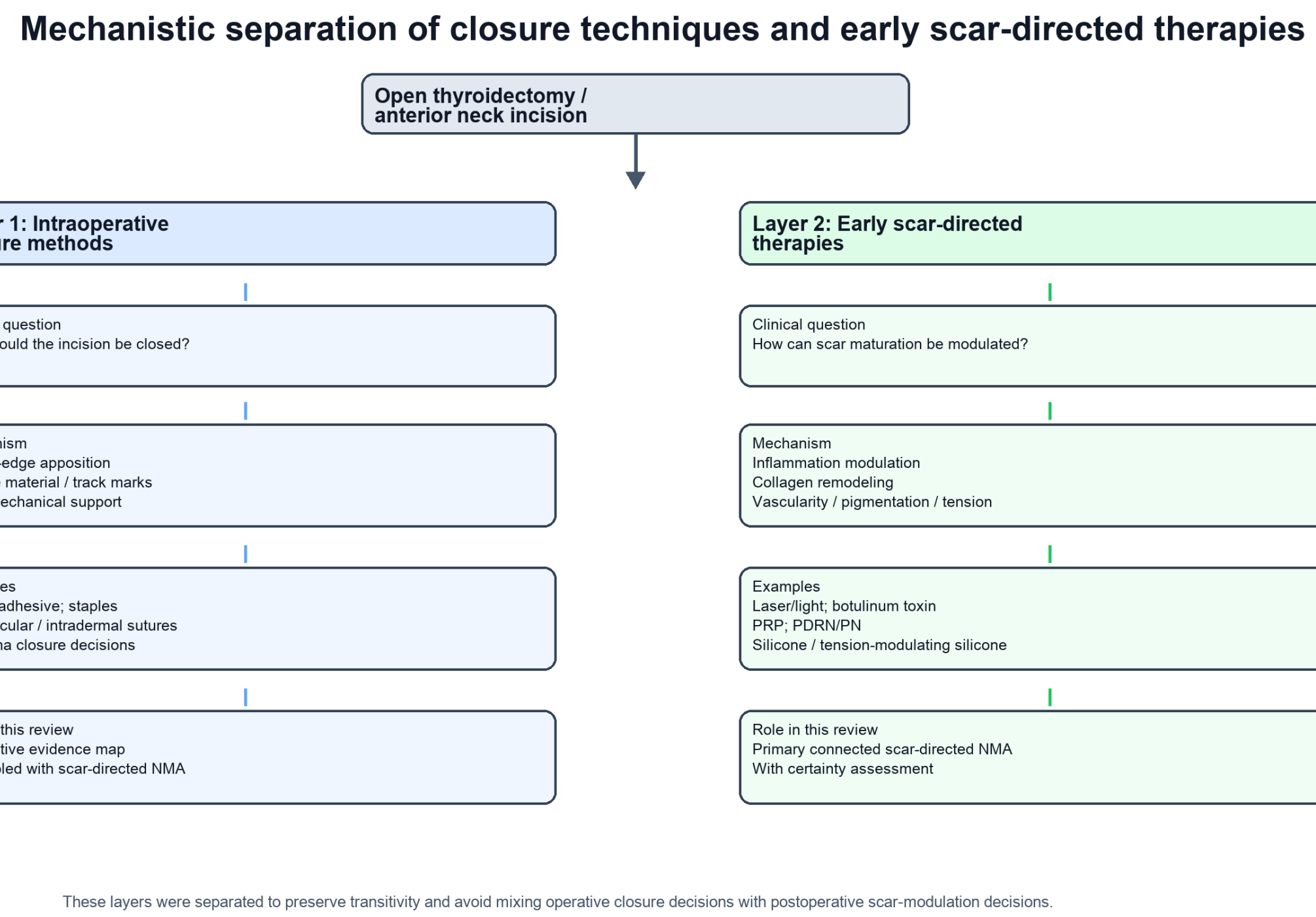

Closure methods were defined as intraoperative wound-edge approximation decisions, whereas early scar-directed therapies were defined as postoperative or perioperative interventions intended to modulate scar biology. These layers were separated before quantitative synthesis to preserve transitivity.
